# Musculoskeletal Modeling and Inverse Dynamic Analysis of Precision Grip in the Japanese Macaque

**DOI:** 10.3389/fnsys.2021.774596

**Published:** 2021-12-08

**Authors:** Tsuyoshi Saito, Naomichi Ogihara, Tomohiko Takei, Kazuhiko Seki

**Affiliations:** ^1^Department of Mechanical Engineering, Faculty of Science and Technology, Keio University, Yokohama, Japan; ^2^Department of Biological Sciences, Graduate School of Science, The University of Tokyo, Tokyo, Japan; ^3^Brain Science Institute, Tamagawa University, Tokyo, Japan; ^4^Department of Neurophysiology, National Institute of Neuroscience, National Center of Neurology and Psychiatry, Tokyo, Japan

**Keywords:** hand, motor control, grasping, optimization, muscle force

## Abstract

Toward clarifying the biomechanics and neural mechanisms underlying coordinated control of the complex hand musculoskeletal system, we constructed an anatomically based musculoskeletal model of the Japanese macaque (*Macaca fuscata*) hand, and then estimated the muscle force of all the hand muscles during a precision grip task using inverse dynamic calculation. The musculoskeletal model was constructed from a computed tomography scan of one adult male macaque cadaver. The hand skeleton was modeled as a chain of rigid links connected by revolute joints. The path of each muscle was defined as a series of points connected by line segments. Using this anatomical model and a model-based matching technique, we constructed 3D hand kinematics during the precision grip task from five simultaneous video recordings. Specifically, we collected electromyographic and kinematic data from one adult male Japanese macaque during the precision grip task and two sequences of the precision grip task were analyzed based on inverse dynamics. Our estimated muscular force patterns were generally in agreement with simultaneously measured electromyographic data. Direct measurement of muscle activations for all the muscles involved in the precision grip task is not feasible, but the present inverse dynamic approach allows estimation for all the hand muscles. Although some methodological limitations certainly exist, the constructed model analysis framework has potential in clarifying the biomechanics and neural control of manual dexterity in macaques and humans.

## Introduction

A complex hand musculoskeletal system enables humans to execute highly coordinated movements, such as firmly grasping and skillfully manipulating objects using the thumb and other fingers. Precision grip is frequently chosen as the subject of experiments focused on the neural control mechanisms underlying human dexterous grip. Precision grip is computationally demanding compared with other grasp types, e.g., power grip. By measuring brain activity during precision grip tasks in macaques, researchers have identified several regions in the central nervous system that contribute to dexterous hand movements. For example, corticospinal projection neurons in the primary motor cortex (e.g., Muir and Lemon, 1983; Bennett and Lemon, 1996; Schieber and Rivlis, 2005) and neurons in the spinal cord (Takei and Seki, 2010, 2013; Alstermark et al., 2011; Takei et al., 2017; Oya et al., 2020) have been implicated in dexterous hand movements.

Precision grip is a very complex mechanical phenomenon. As a result, the relationship between neural activities and the generation of finger force is difficult to define, and can be counterintuitive (Wannier et al., 1991; Maier et al., 1993). For example, cortico-motoneuronal cells in the primary motor cortex, which have an excitatory output effect on finger muscles, are sometimes negatively correlated with the exerted finger force even though their target muscles are positively correlated (Maier et al., 1993). Therefore, an understanding of the dynamic and complex interactions between the nervous system and musculoskeletal system is essential for clarifying the neural mechanisms of primate hand control.

Inverse dynamics analysis is a useful method for investigating the dynamic interactions between the neural system and the musculoskeletal system (Chan and Moran, 2006; Cheng and Loeb, 2008; Jindrich et al., 2011). In this method, muscle forces are estimated by entering motion data into equations describing the motion of a mechanical body system (Erdemir et al., 2007). Such biomechanical modeling and inverse dynamic analyses have (a) been successfully used to describe reaching movements in macaques (Chan and Moran, 2006; Cheng and Loeb, 2008; Jindrich et al., 2011), (b) been useful in distinguishing intrinsic, extrinsic, kinematic, and kinetic variables (Chan and Moran, 2006), and (c) improved the interpretation of kinematic and electrophysiological data (Cheng and Loeb, 2008). However, to the best of our knowledge, no researchers have attempted to construct a hand musculoskeletal model for biomechanical analysis of precision grip in macaques, although musculoskeletal models of the human hand have been developed recently (e.g., Lee et al., 2015; Sachdeva et al., 2015; Mirakhorlo et al., 2018).

In the present study, we constructed an anatomically based, 3D musculoskeletal model of the Japanese macaque hand and biomechanically analyzed macaque’s precision grip based on inverse dynamic calculation, toward clarifying the biomechanics and neural mechanisms underlying coordinated control of the complex hand musculoskeletal system. Specifically, we tested the hypothesis that the inverse dynamic analysis would enable estimation of all the hand muscles involved in the precision grip task. The primate hand possesses an especially complex musculoskeletal structure with at least 39 muscles (Tubiana, 1981), many of which are very difficult to access during *in vivo* experiments. Thus, a complete hand musculoskeletal model could be useful in describing hand muscle activities related to precision grip control. Such research could lead to a deeper understanding of how muscle activities are coordinated within the nervous system for effective control of the complex hand musculoskeletal system.

## Materials and Methods

### Hand Musculoskeletal Model

We constructed a 3D musculoskeletal model of the Japanese macaque (*Macaca fuscata*) hand from a computed tomography scan of one adult male cadaver. The deceased macaque used to be a performing monkey and died naturally as a result of acute respiratory tract infection after living in retirement for more than 10 years. The body mass at the time of death was ∼10 kg. This cadaver had previously been CT scanned to construct a whole-body musculoskeletal model for locomotion studies (Ogihara et al., 2009), but for the present study, we rescanned the cryonically preserved right hand and forearm at a higher resolution (0.096 × 0.096 × 0.095 mm) using a micro CT scanner (ScanXmate-A080S, Comscantecno, Yokohama, Japan). Consecutive cross-sectional images were then transferred to medical imaging software (Analyze 9.0; Biomedical Imaging Resource Mayo Clinic, Rochester, MN, United States) and the 3D surface models of each of the bones were generated as a triangular mesh model using the marching cube method. The experiments took place at the Laboratory of Physical Anthropology, Kyoto University. The use of CT images contributes to reduce the errors of estimated joint moments and muscle forces because it contributes to correct identifications of the locations of joint centers and the orientations of the axes of joint rotations.

We defined the bone coordinate systems embedded in each of the metacarpals (MC) or phalanges (P) using the bone principal axes, the origins of which were located at the centroid, with the *z*-axis as the major axis corresponding to the orientation of the shaft, and the *x*- and *y*-axes corresponding to the radial-ulnar and extension-flexion rotational axes, respectively. Carpal bones were treated as a single mass, ignoring inter-carpal mobility. The origin of the carpal coordinate system was set at the centroid, with the *x*-, *y*-, and *x*-axes facing the dorsal, ulnar, and distal directions, respectively.

**FIGURE 1:**
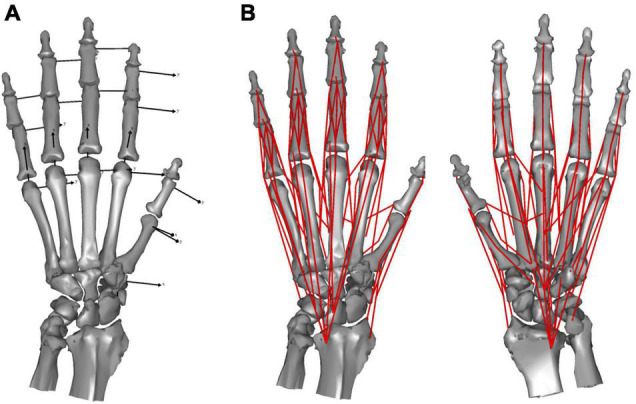
Skeletal model **(A)** and musculoskeletal model **(B)** of the Japanese macaque hand.

We modeled the hand skeleton as a chain of 20 rigid-body bone segments connected by revolute joints. We modeled the interphalangeal (IP) joint as a hinge joint with 1 degree-of-freedom (DOF), capable only of flexion and extension. We approximated the distal articular surface of the phalanges with a cylinder to estimate the joint center. The hinge axis was defined as parallel to the distal *y*-coordinate axis. The metacarpophalangeal (MCP) joints were modeled as joints with 2 DOF, capable of flexion and extension, and radial and ulnar deviation (a universal joint). We approximated the distal articular surface of the metacarpals with a sphere to estimate the joint center. The two rotational joints were assumed to correspond to the *x*- and *y*-axes of the proximal phalanx. The saddle-shaped 1st carpometacarpal (CMC) joint was approximated by a paraboloid surface and modeled as a universal joint with 2 DOF, capable of flexion and extension, and radial and ulnar deviation (Ogihara et al., 2009; Matsuura et al., 2010). The joint center was assumed to be at the apex of the paraboloid and the axes were determined based on the orientation of the paraboloid. The remaining four CMC joints were represented as gimbal joints (joints with three rotational axes intersecting one another), with the joint locations defined as the center of the articular surface. The mobility of CMC joints 2–5 is negligible during precision grip. Thus, for the purposes of this study we treated these and the carpal segment as a single segment. The kinematic skeleton of the macaque hand was thus mathematically described as an open chain of 16 rigid links connected by revolute joints (Figure 1A).

We calculated the mass and the principal moment of inertia for each phalangeal segment as in Ogihara et al. (2009). Briefly, the skin surface model of the hand was divided into phalangeal segments, and the inertial parameters were numerically calculated using each segmented surface model, assuming homogeneous segment composition and a density of 1.0 g/cm^3^ (Table 1). The mass and the principal moment of inertia of the metacarpal and carpal segments were calculated by approximating the corresponding volumes with rectangular solids. The principal axes of inertia of each segment were assumed to be coincident with the bone principal axes, and the center of mass was approximated to be at the origin of the bone fixed-coordinate system.

**TABLE 1:** Inertial parameters for Japanese macaque hand segments.

	Mass (g)	Principal moment of inertia (g mm^2^)		
		Ix	Iy	Iz
Carpus	16.50	2.05.E+03	1.41.E+03	2.36.E+03
1MC	4.70	2.55.E+02	3.73.E+02	1.96.E+02
2MC	6.68	6.77.E+02	8.44.E+02	2.78.E+02
3MC	7.42	9.13.E+02	1.10.E+03	3.09.E+02
4MC	7.10	8.05.E+02	9.82.E+02	2.96.E+02
5MC	6.16	5.38.E+02	6.92.E+02	2.57.E+02
1PP	1.31	4.90.E+01	3.87.E+01	2.15.E+01
1DP	0.43	5.22.E+00	3.41.E+00	4.13.E+00
2PP	2.00	8.18.E+01	9.67.E+01	3.90.E+01
2MP	0.60	9.92.E+00	9.18.E+00	4.89.E+00
2DP	0.43	5.40.E+00	4.50.E+00	3.05.E+00
3PP	3.79	2.37.E+02	2.86.E+02	1.16.E+02
3MP	0.85	2.04.E+01	2.17.E+01	7.80.E+00
3DP	0.68	1.09.E+01	1.20.E+01	6.09.E+00
4PP	3.60	2.13.E+02	2.55.E+02	9.96.E+01
4MP	0.95	2.55.E+01	2.37.E+01	9.37.E+00
4DP	0.62	1.01.E+01	1.04.E+01	4.85.E+00
5PP	2.62	9.63.E+01	1.05.E+02	7.31.E+01
5MP	0.54	9.11.E+00	8.10.E+00	4.15.E+00
5DP	0.41	5.02.E+00	4.38.E+00	2.75.E+00

**FIGURE 2:**
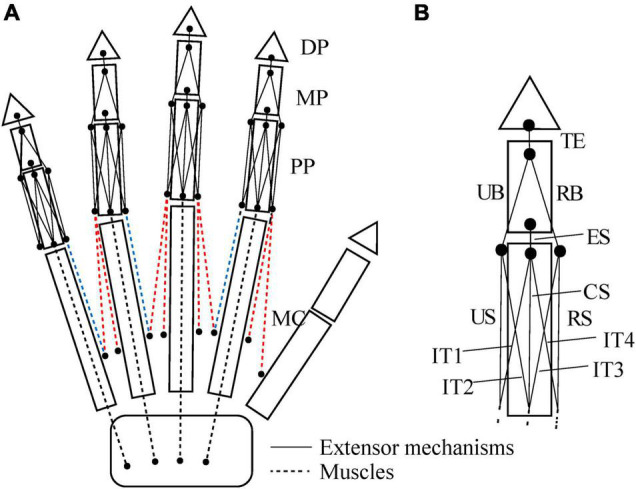
Schematic diagram showing how the interosseous and extensor muscles are connected to extensor mechanisms in this model **(A)**. Extensor mechanisms of the index finger **(B)**. TE, terminal extensor tendon; UB, ulnar band; RB, radial band; ES, extensor slip; US, ulnar slip; CS, central slip; RS, radial slip; IT, interosseous tendon. Tensions of the TE, UB, RB and ES, *f*_*TE*_, *f*_*UB*_, *f*_*RB*_, and *f*_*ES*_ are calculated as follows: *f*_*TE*_ = *f*_*RB*_ + *f*_*UB*_; *f*_*RB*_ = 0.167 × *f_*EDC*2_*; *f*_*UB*_ = 0.167 × *f_*EDC*2_* + 0.333 × *f_1*PIO*_*; *f*_*ES*_ = 0.167 × *f_*EDC*2_* + 0.333 × *f_1*PIO*_* + 0.333 × *f_1*DIOr*_* + 0.333 × *f_1*DIOu*_*. Radial and ulnar components of the 1DIO inserting into the extensor mechanism are denoted by *r* and *u*, respectively.

We defined the muscle-tendon path of each of the 23 hand muscles listed in Table 2 as a series of points connected by line segments (Figure 1B) as in previous studies (e.g., Mirakhorlo et al., 2018; Michaud et al., 2021). Each point, fixed to a corresponding bone coordinate system, was obtained based on our dissection records of the Japanese macaque hand (Ogihara and Oishi, 2012). Origin and insertion points were digitized at the estimated centroids of muscle attachment sites. Intermediate points were determined to properly constrain the muscle-tendon path. Therefore, the muscle-tendon paths do not slide on the bones in the present model. Although there have been some attempts to model such sliding phenomena based on continuum mechanics (e.g., Sachdeva et al., 2015), such complex models usually require additional parameters which possibly introduce additional uncertainties in the model. In addition, previous studies suggested the slight changes in the muscle paths did not markedly affect the results of the simulations (Valente et al., 2014; Kainz et al., 2021). Hence, each muscle-tendon path was represented as a series of points connected by line segments. We modeled muscles that are divided or split along the muscle path, such as the FDP, FDS, and EDC (see Table 2 for abbreviations), as groups of several independent muscle paths. Maximum muscle force was assumed to be proportional to the physiological cross-sectional area (PCSA), and was calculated by multiplying the PCSA by the maximum muscle stress (23 N/cm^2^) (Spector et al., 1980) as in Delp et al. (1990) and Lemay and Crago (1996), although no study has validated that the muscle stress value measured from cat soleus (Spector et al., 1980) can be applicable to primate hand muscles. The PCSA of each muscle was obtained from Macaque A in Ogihara and Oishi (2012) since this was the same macaque that had been CT scanned for the construction of the present model. We set the PCSA of the FDP1 and CD2 to zero as these muscles are absent in the Japanese macaque (Ogihara and Oishi, 2012). Pennation angle was not considered in the calculation because this value is typically very small for hand muscles and thus is not expected to significantly affect the force-generating capacity of the muscles. The extensor mechanism (dorsal aponeurosis) on the dorsal side of each long finger was modeled as a net structure, based on the methods of Garcia-Elias et al. (1991) and Sancho-Bru et al. (2001). We determined the load transmission of muscles to the extensor mechanism based on the methods of Chao et al. (1989). Figure 2 illustrates how the extensor mechanisms were connected with the interosseous and extensor digitorum muscles to generate torques around the IP joints. We did not include lumbrical muscles in this model because the force generating capacity of these muscles is very small. In addition, lumbrical muscles are difficult to emulate because they originate from the FDP tendons, but are not directly connected to a bone segment.

**TABLE 2:** Muscle parameters for the Japanese macaque hand.

	muscle name	Abb	PCSA (cm^2^) max.	force (*N*)
1	Flexor digitorum superficialis	FDS		
	FDS2	FDS2	0.45	10.4
	FDS3	FDS3	0.85	19.6
	FDS4	FDS4	0.95	21.9
	FDS5	FDS5	1.23	28.3
2	Flexor digitorum profundus	FDP		
	FDP1	FDP1	0.00	0.0
	FDP2	FDP2	1.81	41.6
	FDP3	FDP3	1.39	32.0
	FDP4	FDP4	1.29	29.7
	FDP5	FDP5	1.63	37.5
3	Extensor digitorum communis	EDC		
	EDC2	EDC2	0.29	6.7
	EDC3	EDC3	0.08	1.8
	EDC4	EDC4	0.12	2.8
	EDC5	EDC5	0.32	7.4
4	Extensor digiti secundi & tertii proprius			
	ED2P	ED2P	0.23	5.3
	ED3P	ED3P	0.08	1.8
5	Extensor digiti quarti & quinti proprius			
	ED4P	ED4P	0.22	5.1
	ED5P	ED5P	0.19	4.4
6	Abductor pollicis longus	ABPL	2.67	61.4
7	Extensor pollicis longus	EPL	0.19	4.4
8	Abductor pollicis brevis	ABPB	0.71	16.3
9	Flexor pollicis brevis	FPB	0.32	7.4
10	Opponens pollicis	OP	0.34	7.8
11	Adductor pollicis	ADP		
	ADP oblique	ADPo	0.17	3.9
	ADP transverse	ADPt	0.43	9.9
12	Abductor digiti minimi	ABDM	1.17	26.9
13	Flexor digiti minimi brevis	FDMB	0.33	7.6
14	Opponens digiti minimi	ODM	0.63	14.5
15	Dorsal interosseous 1 radial head	1DIOr	0.52	12.0
	Dorsal interosseous 1 ulnar head	1DIOu	0.52	12.0
16	Dorsal interosseous 2 radial head	2DIOr	0.40	9.1
	Dorsal interosseous 2 ulnar head	2DIOu	0.40	9.1
17	Dorsal interosseous 3 radial head	3DIOr	0.30	6.9
	Dorsal interosseous 3 ulnar head	3DIOu	0.30	6.9
18	Dorsal interosseous 4 radial head	4DIOr	0.23	5.3
	Dorsal interosseous 4 ulnar head	4DIOu	0.23	5.3
19	Palmar interosseous 1	1PIO	0.56	12.9
20	Palmar interosseous 2	2PIO	0.18	4.1
21	Palmar interosseous 3	3PIO	0.24	5.5
22	Contrahens digiti quarti	CD4	0.21	4.8
23	Contrahens digiti quinti	CD5	0.22	5.1
	Contrahens digiti scundi	CD2	0.00	0.0

*Abb, abbreviation; PCSA, physiological cross-sectional area of muscle.*

Our anatomically accurate model of the Japanese macaque hand was entered into the musculoskeletal simulation software package Anybody Modeling System (AnyBody Technology, Aalborg, Denmark) (Damsgaard et al., 2006). This software is capable of constructing a musculoskeletal model using a programming language (Anyscript) and the inverse dynamic analysis of the developed musculoskeletal system.

### Precision Grip Task

We collected electromyographic (EMG) and kinematic data from one adult male Japanese macaque (age, 8 years, body weight, 8.0 kg) during the precision grip task. The experiments took place at the Department of Neurophysiology, National institute of Neuroscience, National Center of Neurology and Psychiatry. The animal was treated in strict compliance with the Animal Care and Use Committee of the National Institute of Neuroscience, and with the NIH Guide for the Care and Use of Laboratory Animals. The protocol was approved by the local ethics committee for primate research at National Institute of Neuroscience (Permit #: 2012-004). All studies were performed in accordance with the recommendations of the Weatherall report, “The use of non-human primates in research.” The animal was housed in a cage (80 × 70 × 80 cm) individually in a large room for the care of macaque monkeys in the primate research facility of the National Institute of Neuroscience, along with several other monkeys permitting rich visual, olfactory and auditory interactions. Water was available *ad libitum*, and a standard commercially formulated non-human primate diet (AS, Oriental Yeast, Tokyo, Japan) was provided once daily (120 g/meal) and supplemented daily with fresh fruit. Some toys (such as balls, empty plastic bottle, etc.) were given to the monkey as part of the environmental enrichment approach. Regular care and monitoring, balanced nutrition and environmental enrichment were provided by the staffs of this center. All surgeries were performed using sevoflurane anesthesia (1.5–2.5% in 2:1 O_2_:N_2_O), and the monkey was artificially respired. Respiration rate was adjusted to keep end-tidal CO_2_ within 30–35 mmHg.

**FIGURE 3:**
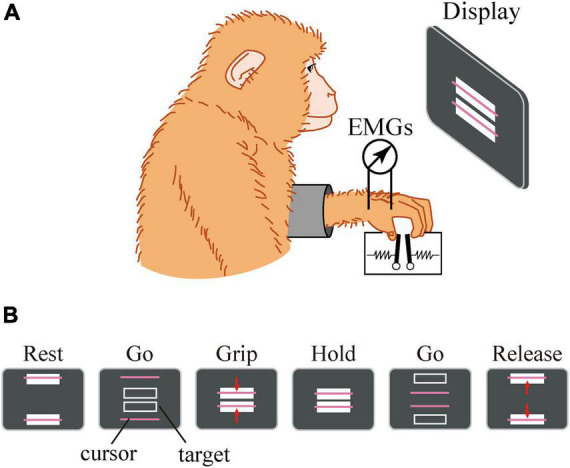
Experimental setup. The macaque sat in front of a computer screen **(A)**. The lever positions were displayed on a computer screen as cursors, and the macaque was required to grip, hold, and release the levers using the thumb and index finger according to the movement of a target displayed on the screen **(B)**.

The details of the precision grip task, experimental setup, surgical operation, and EMG recording procedure were similar to those described previously (Takei and Seki, 2008). Briefly, the macaque monkey has been trained to grip, hold, and release spring-loaded levers using the thumb and index finger. The monkey sat in a chair with its right and left elbow restrained, and the thumb and index finger were inserted to separate holes to access the levers. Therefore, the position of the hand and fingers were quite stationary during the precision grip task. The lever positions were displayed on a computer screen as cursors, and the macaque was required to grip, hold, and release the levers according to the movement of the target displayed on the screen (Figure 3). The reaction forces applied to the fingertips were measured by strain gauges attached to the pinch levers. For EMG recording, pairs of stainless steel wires (AS631, Cooner Wire) were chronically implanted subcutaneously in the following eight muscles: (1) 1DIO, (2) FDS2, (3) FDP2, (4) ADP, (5) ABPL, (6) EDC2, (7) ED2P, and (8) ABPB. The EMG signals were amplified and filtered using a multichannel differential amplifier (5–3000 Hz; SS-6110, Nihon Kohden, Japan) and digitized at 5 kHz. Simultaneously, signals from the strain gauges were digitized at 1 kHz. Offline, the EMG signals were high-pass filtered (cut-off = 30 Hz), rectified, and downsampled to 100 Hz. The signal was then low-pass filtered (cut-off = 2.5 Hz) to obtain a linear envelope. The signals from the strain gauges were low-pass filtered (cut-off = 10 Hz) and downsampled to 100 Hz.

Simultaneous to the EMG recording, we filmed the hand movements of the macaque during the precision grip task using five AVCHD video cameras with a resolution of 1920 × 1080 (iVIS HF S21, Canon, Tokyo, Japan). From the motion images, we extracted well-recorded precision grip sequences and manually digitized the following 12 positions frame-by-frame: (1) tip of the 1DP, (2) head of the 1PP, (3) head of the 1MC, (4) 1CMC joint, (5) tip of the 2DP, (6) head of the 2MP, (7) head of the 2DP, (8) head of the 2MC, (9) head of the 3MC, (10) head of the 4MC, (11) head of the 5MC, and (12) styloid process of the ulna. The positions of the third, fourth, and fifth fingers were not digitized as these fingers were deeply flexed such that the joint positions were not detected by the cameras. We calculated the 3D movements of the 12 digitized positions using WinAnalyze 3D motion analysis software (Mikromak, Berlin, Germany) and digitally low-pass filtered the data at 5 Hz. The standard error of the mean accompanying each of the measured positions in the calibration was about 1.4 mm. The change in position of each coordinate over time was approximated by a polynomial function.

To replicate the 3D musculoskeletal kinematics of the macaque hand during the precision grip task, the model was first scaled to the size of the filmed macaque hand based on the length of the index finger. The hand musculoskeletal model was then registered to the recorded motion data by means of a least squares approach, i.e., minimizing the sum of distances between each digitized coordinate and the corresponding point on the hand model. The error associated with the scaling of a generic model has been suggested to be relatively minor (Valente et al., 2014; Kainz et al., 2021).

### Inverse Dynamic Analysis of Precision Grip

Using the inverse dynamic method, the net joint torques required to perform a given motion can be uniquely computed from the measured kinematics and external forces, i.e., the lever forces in the present study. However, joint torques can be achieved by an infinitely large number of combinations of muscle forces, as the number of muscles spanning a joint far exceeds the DOF of the corresponding joint. In this study, we addressed the indeterminant redundancy problem of muscle recruitment using an optimization method (Tsirakos et al., 1997). We used the sum of the cubes of muscle stress, which are considered to be a proxy for muscle fatigue and hence a physiologically relevant measure, as an objective function (Crowninshield and Brand, 1981). Such calculation was conducted using Anybody Modeling System (AnyBody Technology, Aalborg, Denmark).

In the present study, the MCP and 1CMC joints were modeled as universal joints. In a universal joint, two components of the joint torque vector (flexion/extension and radial/ulnar deviation torques) should be simultaneously balanced by the sum of moments generated by the muscles spanning the joint. However, this was actually difficult because joint constraints due to passive elastic elements such as collateral and carpometacarpal ligaments and joint capsules were not imposed in the present model. Therefore, when conducting the inverse dynamic analysis, the rotational axes of the radio-ulnar deviation of the MCP joints and the radio-ulnar deviation and flexion/extension axes of the 1CMC joints were assumed to be instantaneously immobile in the present study, hence the respective torques were structurally balanced by reaction torques around each rotational axis.

To compare the predicted muscular force profiles with the shape and timing of the enveloped EMG signals, we calculated the cross-correlation *r* with zero time-lag (Blana et al., 2008; Michaud et al., 2021). The cross-correlation is a measure of the similarity between two waveforms, represented as a scalar between −1 and 1. If the two waveforms are identical, *r* is equal to 1. If the amplitude of the EMG signal was effectively zero, or the maximum force of the predicted muscular force was less than 0.3 N, we did not calculate the cross-correlation.

## Results

**FIGURE 4:**
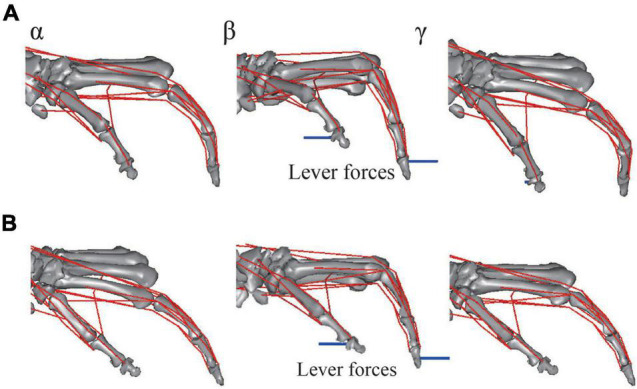
Graphical representation of reconstructed hand musculoskeletal kinematics of the Japanese macaque during the precision grip task. **(A)** Trial 1. **(B)** Trial 2. α, β, and γ correspond to the moments at which the levers had just been gripped (α), the maximum forces had been applied by the index finger (β), and the levers had been released (γ), respectively. Red lines, muscle paths; blue lines, lever forces. Joint angles were defined as positive for extension and adduction of the 1CMC and MCP joints and for extension of the IP joints. Joint angles were defined as zero at *t* = 0.

**FIGURE 5:**
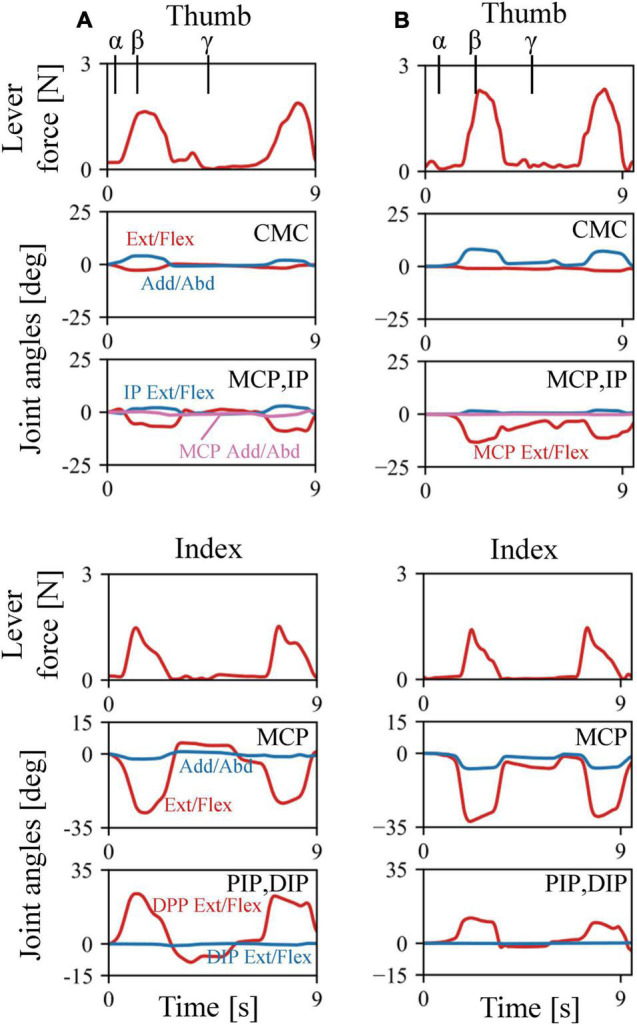
Changes in lever forces and joint angles during the precision grip task in the Japanese macaque. **(A)** Trial 1. **(B)** Trial 2. Arrows α, β, and γ correspond to the moments at which the levers had just been gripped (α), the maximum forces were applied by the index finger (β), and the levers were released (γ), respectively.

The musculoskeletal architecture of the macaque hand was well emulated by the computer model such that the 3D hand kinematics during precision grip were successfully reconstructed and visualized (Figure 4). The 3D hand musculoskeletal kinematics for two sequences of the precision grip task in the Japanese macaque were presented in Figure 4, and α, β, and γ correspond to the moment at which the lever had just been gripped (α), the moment at which the maximum force (blue line) had been applied by the index finger (β), and the moment at which the lever was released (γ). The mean matching bias of the markers (distance between each motion-captured marker and the corresponding marker on the model) averaged over time was 0.67 ± 0.03 and 0.47 ± 0.04 mm (mean ± SD) for the first and second sequences, respectively, indicating that the matching between the hand model and the digitized coordinates was reasonably high. We analyzed only two sequences because the hand and finger movements were quite stereotypical in the precision grip task (see section “Materials and Methods”) and hence the variability of the movements was small in the present study. The peak force applied by the fingers was ∼1.5 N, and a comparatively larger force was applied by the thumb (∼2 N) during the precision grip task (Figure 5). It is also observed that the CMC, MCP, and IP joints of the thumb were mainly adducted, flexed, and extended, respectively, and the MCP and PIP joints of the index finger were mainly flexed and extended, respectively, when the lever was gripped (Figure 5).

**FIGURE 6:**
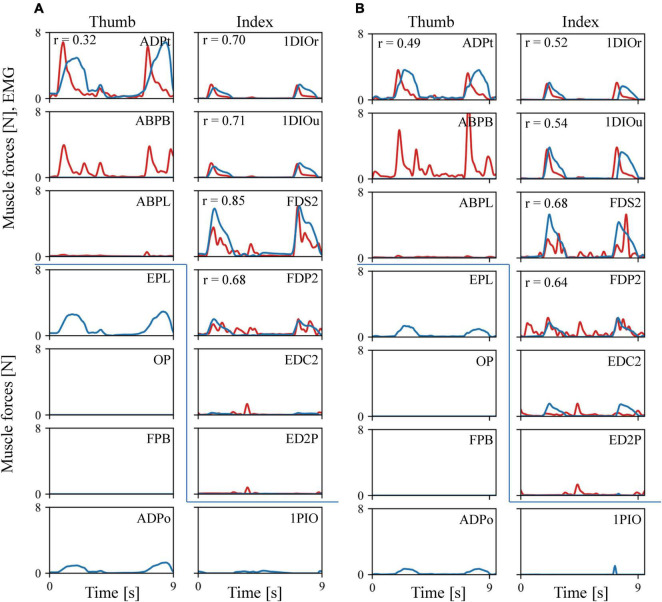
Comparisons between the recorded EMG (red line) and the predicted muscle force patterns (blue line). **(A)** Trial 1. **(B)** Trial 2. As EMG amplitude is not directly comparable to the predicted muscular force, we scaled the EMG amplitude by the maximum force of the corresponding muscle. If the maximum force was less than 0.3N, or the amplitude of the EMG signal is effectively zero, the EMG amplitude was adjusted such that 100 mV was equal to 1 N.

The estimated muscular force patterns during the precision task were quite similar to the corresponding EMG patterns (Figure 6). Our inverse dynamic analysis estimated that the 1DIOr, 1DIOu, ADPt, FDS2, and FDP2 muscles would be activated when the levers were pinched, and that muscle activity would be negligible in the ABPL, EDC2, and ED2P muscles during the precision grip task. The cross-correlation values were generally greater than 0.6 for the 1DIOr, 1DIOu, FDS2, and FDP2 muscles. In these muscles, the peaks of the muscle forces correspond well to those of the EMG signals. Furthermore, the amplitudes of the EMG signal were effectively zero in the ABPL, EDC2, and ED2P muscles, muscle forces of which were estimated to be negligible. Therefore, the estimated muscular forces were generally in accordance with the EMG signals for six out of eight muscles. However, although the amplitude of the measured EMG signal was quite large in the ABPB muscle, the estimated muscular force pattern was zero in the present study. In addition, the cross-correlation values to be relatively low for the ADPt, indicating that the prediction was less accurate for these muscles.

Our model also predicted the force induced by muscles for which we did not obtain an EMG signal during the experiments (Figure 6). For example, the EPL and ADPo were predicted to be active when the levers were pinched, and no activity was predicted for the FPB or OP.

## Discussion

We used a newly constructed anatomically realistic musculoskeletal hand model to biomechanically analyze a precision grip task in the Japanese macaque using inverse dynamic calculations. Because of the structural complexity of the primate hand, this topic has challenged researchers in the field of biomechanics. As a result, few studies have succeeded in using the inverse dynamic approach to analyze hand movements even in humans (Grinyagin et al., 2005; Goislard de Monsabert et al., 2012; Wu et al., 2012). Additionally, to the best of our knowledge, no studies have compared calculated muscle force profiles with simultaneously recorded EMG data, as it is generally impractical to record EMG signals from multiple human hand muscles *in vivo*. Thus, the present study represents the first attempt to compare calculated force profiles of hand muscles with EMG signals recorded during a precision grip task with the intention of evaluating the extent to which inverse dynamic analysis (based on an anatomically precise musculoskeletal model) can predict muscle activities during hand movements.

Our results demonstrate that optimized muscle forces calculated using the inverse dynamic approach are similar to those obtained by recording EMG signals during the precision grip task. One advantage of the inverse dynamic approach is that the muscle forces or activation levels can be estimated for all the muscles involved in the precision grip task. Placing wire electrodes in all of the hand muscles *via* a surgical intervention is generally difficult, even for non-human primates. However, if muscular force predictions based on the inverse dynamic approach are reasonably accurate, as in the present study, this technique may be applied to analyses of human precision grip capability.

We found that the 1DIO, ADPt, ADPo, and EPL were active during the precision grip task in the Japanese macaque. Although direct comparisons between macaques and humans are not possible owing to differences in hand anatomy and the kinematics of precision grip, our predicted muscle activation patterns were generally in agreement with EMG activity patterns recorded during the human precision grip task (Long et al., 1970; Cooney et al., 1985). However, we found the OP muscle to be inactive during precision grip, even though it is known to be active in humans (Long et al., 1970; Cooney et al., 1985) and also in macaques (Smith and Bourbonnais, 1981). This is possibly because the macaque used the ulnar side of the thumb, instead of the pulp, to push the lever during the precision grip task. Therefore, it is likely that the OP, which functions to oppose the thumb with the other fingers, was not activated during the precision grip task. Furthermore, we predicted that the EPL would be active during the hold phase. This is consistent with EMG recordings obtained during the precision grip task in humans (Cooney et al., 1985; Rufener and Hepp-Reymond, 1988) as well as macaques (Smith and Bourbonnais, 1981). In the precision grip task, activation of the extensor muscle is probably necessary for fine adjustments to the direction of fingertip force.

Despite our compelling results, our prediction of muscle activation was certainly not perfect. Particularly, the agreement between the force and EMG patterns was not sufficient for the ABPB and ADPt muscles. This discrepancy may be attributable to inaccuracies in the mechanical modeling of the hand musculoskeletal system. Although we attempted to construct the model to be anatomically realistic, the hand of a CT-scanned and dissected macaque is not identical to that of a motion-captured macaque. In addition, the present study assumed that the fingertip forces were horizontal and parallel to the direction of the lever movements as each fingertip force were measured by a uniaxial transducer, but there might have been vertical and mediolateral force components that possibly affected the prediction of the muscle forces. Furthermore, we did not consider the influence of passive elastic elements around the joints or certain muscle properties, such as force-length and force-velocity relationships. This is owing to the fact that such joint and muscle properties of the hand in the macaque have not been quantitatively investigated, but such simplifications may have affected the accuracy of our muscle force predictions. Further improvements to the accuracy of the model are necessary for improved muscle force prediction in the precision grip task. Conversely, the discrepancy could have arisen from the measurement of the EMG signals. For instance, EMG data generated using fine wires that are implanted within muscles can represent only a fraction of all active motor units in the target muscle (Basmajian and De Luca, 1985).

Of the existing limitations of this study, the muscle redundancy problem is the most vital (Tsirakos et al., 1997). In the present study, the sum of the cubes of muscle stress was chosen as an objective function for solving the redundancy problem of muscle recruitment. However, the way in which the central nervous system solves this redundancy problem remains unclear. Indeed, our computational technique would be improved by incorporating more biologically feasible hypotheses about this issue. For example, muscle synergies (i.e., co-activation of muscles by a single neural signal) may facilitate the control of redundant degrees-of-freedom in the musculoskeletal system. Spinal interneurons could form the structural basis for functional muscle synergies (for a review, see Bizzi et al., 2008). The ability to incorporate neuronal constraints related to muscle synergies into our inverse dynamic analysis could increase the accuracy of muscle force estimations. This, in turn, could lead to new hypotheses about how the nervous system solves the redundancy problem of muscle recruitment (Moritani and Ogihara, 2019). Therefore, the present model-based analysis of neurophysiological data may serve as a viable framework for understanding how the central nervous system deals with muscle recruitment redundancy, one of the central unresolved issues in the fields of neuroscience and biomechanics. Forward dynamic simulation of the precision grip based on the present hand model can also be used for the same purpose and should also be investigated in future studies (Gustus et al., 2012; Moritani and Ogihara, 2019).

## Data Availability Statement

The raw data supporting the conclusions of this article are available by the authors upon reasonable request.

## Ethics Statement

The animal study was reviewed and approved by the local ethics committee for primate research at National Institute of Neuroscience (Permit #: 2012-004).

## Author Contributions

NO and KS conceived and designed the study. TS and NO constructed the musculoskeletal model and drafted the manuscript. TS, NO, TT, and KS performed the experiment and data analysis. All authors edited and approved the manuscript prior to submission.

## Conflict of Interest

The reviewer SS declared a shared affiliation with one of the authors NO at the time of review. The authors declare that the research was conducted in the absence of any commercial or financial relationships that could be construed as a potential conflict of interest.

## Publisher’s Note

All claims expressed in this article are solely those of the authors and do not necessarily represent those of their affiliated organizations, or those of the publisher, the editors and the reviewers. Any product that may be evaluated in this article, or claim that may be made by its manufacturer, is not guaranteed or endorsed by the publisher.
